# Effect of immune cells and plasma metabolites on inflammatory bowel disease: Two-sample Mendelian randomization study and mediation analysis

**DOI:** 10.1097/MD.0000000000046116

**Published:** 2025-11-28

**Authors:** Zhong Li, Haiyan Wen, Xueyun Huang, Na Wu

**Affiliations:** a Department of Geratology, Affiliated Hospital of Jiangxi University of Chinese Medicine, Nanchang, Jiangxi Province, China; b Department of Postgraduate, Jiangxi University of Chinese Medicine, Nanchang, Jiangxi Province, China; c Department of Gastroenterology, Affiliated Hospital of Jiangxi University of Chinese Medicine, Nanchang, Jiangxi Province, China.

**Keywords:** immune cell, inflammatory bowel disease, mediation analyses, Mendelian randomization, plasma metabolite

## Abstract

Inflammatory bowel disease (IBD) represents an immune-related inflammation of the gastrointestinal tract. Current research highlights the significant role of immune mechanisms and metabolic components on its development and progression. Nonetheless, the interplay between these factors remains inadequately explored. This study used Mendelian randomization (MR) to analyze the causality between immune cells, plasma metabolites, and IBD, and to identify potential mediation metabolites. This study used 2-sample MR analysis method to assess the associations between 731 immune cell types, 1400 plasma metabolites, and IBD, and subtypes ulcerative colitis (UC) and Crohn’s disease (CD). The inverse-variance weighted, Simple mode, Weighted median, and Weighted mode approaches confirmed the validity of the results. Horizontal pleiotropy was evaluated via MR-Egger regression, heterogeneity through Cochran’s *Q* test, and sensitivity using the leave-one-out method. Mediation analysis pinpointed potential mediation metabolites linking immune cells with IBD and its subtypes. MR analysis identified causal associations involving 17 immune cell phenotypes and 37 metabolites with IBD, 13 immune cell phenotypes and 15 metabolites with UC, and 11 immune cell phenotypes and 19 plasma metabolites with CD (*P* < .01). Mediation analysis demonstrated that the association between CD25 on IgD+ CD38br and IBD was mediated by the arachidonate (20:4n6) to oleate to vaccenate (18:1) ratio, with a mediation proportion of 7.42%. CD28+ CD45RA+ CD8dim absolute cell and HLA DR on B cells mediated the associations between 3-(4-hydroxyphenyl) lactate, carnitine C14 levels, and UC, with mediation proportions of 6.97% and 8.8%, respectively. CD27 on sw mem and CD3 on EM CD4+ mediated the associations between mannonate levels, 2-hydroxy-4-(methylthio) butanoic acid levels, and CD, with mediation proportions of 10.9% and 5.89%, respectively. The study demonstrates the causal association of immune cells and plasma metabolites with IBD, UC, and CD, providing novel insights in terms of the prevention, diagnosis, and management of these conditions.

## 1. Introduction

Inflammatory bowel disease (IBD) encompasses idiopathic inflammations affecting the ileum, rectum, and colon, with subtypes being primarily ulcerative colitis (UC) and Crohn’s disease (CD).^[1]^ Patients typically experience recurrent diarrhea, abdominal pain, and bloody stools. This refractory condition exhibits a chronic, lifelong relapsing course, imposing substantial socioeconomic and psychological burdens.^[2]^ According to the global burden of disease study, the number of prevalent IBD cases increased by 1,75,904 and the incidence rate rose by 88.30% from 1990 to 2021.^[3]^ The etiology of IBD remains incompletely understood, currently attributed to interactions among environmental, genetic, infectious, and immunological factors. Abnormal activation of the intestinal mucosal immune response is crucial in IBD pathogenesis.^[4]^ The normal immune system maintains homeostasis by distinguishing self from nonself and eliminating foreign antigens through immune responses. External antigen invasion or colonic epithelial damage triggers immune responses, engaging various immune cells in the pathogenesis of IBD.^[5]^ Substantial experimental evidence identifies abnormalities in immune cells such as CD4, CD25, DC, and HLA DR in IBD patients.^[6]^

Plasma are small-molecule compounds generated or utilized in various metabolic processes, essential for maintaining intestinal barrier integrity and immune balance by nourishing epithelial cells and directly or indirectly activating various receptors.^[7]^ IBD patients often display abnormal levels of metabolites such as valine, tryptophan, niacin, and taurine.^[8–11]^ These dysregulated metabolites during intestinal inflammation may initially trigger IBD onset. Research indicates that the impact of immune cells on disease progression can be influenced by multiple factors, including metabolites. For instance, in tumors, oncometabolites play a crucial regulatory role in the interplay between tumor cells and immune cells, which can influence tumor growth and the immune evasion capacity of malignant cells.^[12,13]^ While associations between immune cells and metabolites with IBD are established, their causal associations and mediation effects remain unclear.

Mendelian Randomization (MR) employed genome-wide association study (GWAS) data and single nucleotide polymorphisms (SNPs) as instrumental variables (IVs) to study causal relationships between exposures and outcomes, effectively excluding reverse causation and confounding factors, thus addressing the limitations of traditional observational studies.^[14]^ The 2-step Mendelian Randomization (TSMR) approach can identify potential mediators between an exposure and an outcome. In this study, we first identified immune cells and plasma metabolites causally associated with IBD, then used TSMR analysis method to explore the mediation effects of plasma metabolites in the relationship between immune cells and IBD.

## 2. Methods and materials

### 2.1. Study design

**Figure 1.**
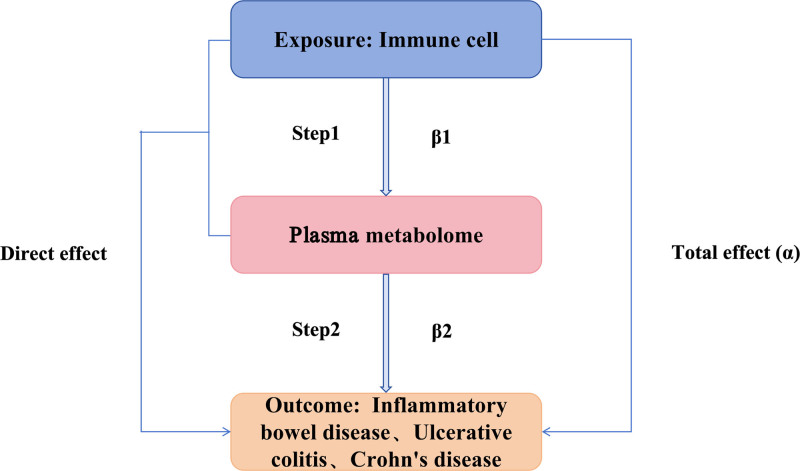
Study flowchart.

A 2-sample MR approach was employed to explore causal associations among immune cells, plasma metabolites, and IBD (UC and CD) using genes as IVs. Published genome-wide summary data for immune cells and plasma metabolites were analyzed to identify statistically significant SNPs serving as IVs. These SNPs were then utilized in various MR methods to assess the causal associations among immune cells, plasma metabolites, and IBD (UC and CD). When using SNPs as IVs to examine causal associations of exposure with outcome, 3 hypotheses must be met^[15]^: IVs must have a strong association with the exposure; IVs should be unrelated to confounders affecting both exposure and outcome; and IVs must impact the outcome exclusively through the exposure, not via alternative pathways. Subsequently, a TSMR was conducted to study mediation role of metabolites between immune cells and IBD (UC and CD; Fig. 1).

### 2.2. Data source

The GWAS dataset of immune cells (https://www.ebi.ac.uk/gwas/; registration numbers ranging from GCST0001391 to GCST0002121)^[16]^ from the European Bioinformatics Institute included 3757 individuals of European descent, approximately 2,40,000 SNPs, and 731 immune phenotypes. These phenotypes spanned 118 absolute cell (AC) counts, 192 relative cell counts, 389 median fluorescence intensities (reflecting surface antigen levels), and 32 morphology parameters, across 6 panels: B cells, CDC, T-cell maturation stage, monocytes, myeloid cells, TBNK (B cells, NK cells, and T-cells), and Treg.

Summary statistical data for plasma metabolomics were sourced from the GWAS catalog (https://www.ebi.ac.uk/gwas/; registration numbers: GCST90199621 to GCST90201020),^[17]^ encompassing 8299 individuals of European descent, and including data on 1400 plasma metabolites – comprising 1091 individual metabolites and 309 metabolite ratios.

The IBD GWAS dataset (ieu-a-31) from the integrative epidemiology unit (IEU) OpenGWAS project included 34,652 participants (12,882 patients and 21,770 controls) and 12,716,084 SNPs. The UC GWAS dataset (ieu-a-32) from the same project included 27,432 participants (6968 patients and 20,464 controls) and 1,22,55,197 SNPs. The CD GWAS dataset (ieu-a-30) also from IEU OpenGWAS included 20,883 individuals (5956 patients and 14,927 controls) and 1,22,76,506 SNPs (Table 1). As these datasets were publicly available, ethical approval was waived.

**Table 1:** Characteristics of summary genome-wide association studies.

Trait	GWAS ID	Sample size	Ancestry
Immune cells	ebi-a-GCST0001391-ebi-a-GCST0002121	3757	European
Plasma metabolomics	GCST90199621~GCST90201020	8299	European
IBD	ieu-a-31	34,652	European
UC	ieu-a-32	27,432	European
CD	ieu-a-30	20,883	European

CD = Crohn's disease, GWAS = genome-wide variance weighted, IBD = inflammatory bowel disease, UC = ulcerative colitis.

### 2.3. Selection of IVs

From the GWAS summary datasets, SNPs qualifying as IVs were identified. An initial correlation threshold of *P* < 1 × 10^−5^ was selected to screen SNPs associated with exposure factors. Then, linkage disequilibrium was excluded using an *r*² < 0.001 and a genetic distance of 10,000 kb.^[18,19]^ To minimize bias from weak IVs, the F statistic was computed as *F* = β²/SE² (with β representing the SNP’s effect size on exposure factors and SE as the standard error of β). SNPs exhibiting *F* statistics <10 were discarded. Allele and effect data between exposure and outcome were then harmonized, with palindromic SNPs being removed. Additionally, ambiguous and duplicate SNPs were removed. Ultimately, the SNPs utilized for MR analysis were finalized.^[20]^

### 2.4. Statistical analysis

#### 2.4.1. MR analysis

The MR analysis employed the “2-Sample MR” package in R 4.2.0.^[21]^ Inverse-variance weighted (IVW) was the principal approach for assessing the causal associations of 731 immune cells and 1400 plasma metabolites with IBD, UC, and CD. This approach weighted the causal effects of various genetic variants on traits and combined these weighted estimates to determine the overall causal effect. Its advantages included reducing sample size impact, improving estimation precision, and minimizing bias, thus establishing it as the main analyses in this study. Additional methods, such as MR-Egger, weighted median, weighted mode, and simple mode, were used to validate the consistency of MR analysis results by calculating the β value, improving the robustness of causal associations. Results were expressed by odds ratios with 95% confidence intervals, and a *P*-value of <.05 suggested a significant association between exposure and outcome. False discovery rate (FDR) correction was performed to account for type I errors arising from multiple testing. An FDR < 0.05 was defined as strong evidence for significance, while FDR > 0.05 but a nominal *P*-value < .05 indicated suggestive evidence of association.

The TSMR method examined the mediating role of plasma metabolites between immune cells and IBD (UC and CD). Initially, a 2-sample MR estimated the effect of immune cells on IBD (UC and CD), determining the total effect (α). Subsequently, TSMR evaluated the impact of immune cells on plasma metabolites (β1) and the influence of plasma metabolites on IBD (UC and CD; β2). The mediation proportion was calculated as (β_1_ * β_2_/α) × 100%, quantifying the mediating role of plasma metabolites in the association between immune cells and IBD (UC and CD).

#### 2.4.2. Sensitivity analysis

This study used various sensitivity analyses to study the validity and robustness of the results. MR-Egger regression tested for horizontal pleiotropy, where a significant intercept term indicated pleiotropy.^[22]^ Cochran’s *Q* test evaluated result heterogeneity, with a *P*-value < .05 suggesting heterogeneity.^[23]^ Sensitivity analysis included the leave-one-out (LOO) method, sequentially removing each SNP and calculating the combined effect of the remaining SNPs. If the MR results of the remaining IVs after removal of a specific IV slightly differed from the total results, it suggested the validity and robustness of the MR analysis results.^[24]^

## 3. Results

### 3.1. MR analysis of immune cells and IBD and its subtypes

**Figure 2.**
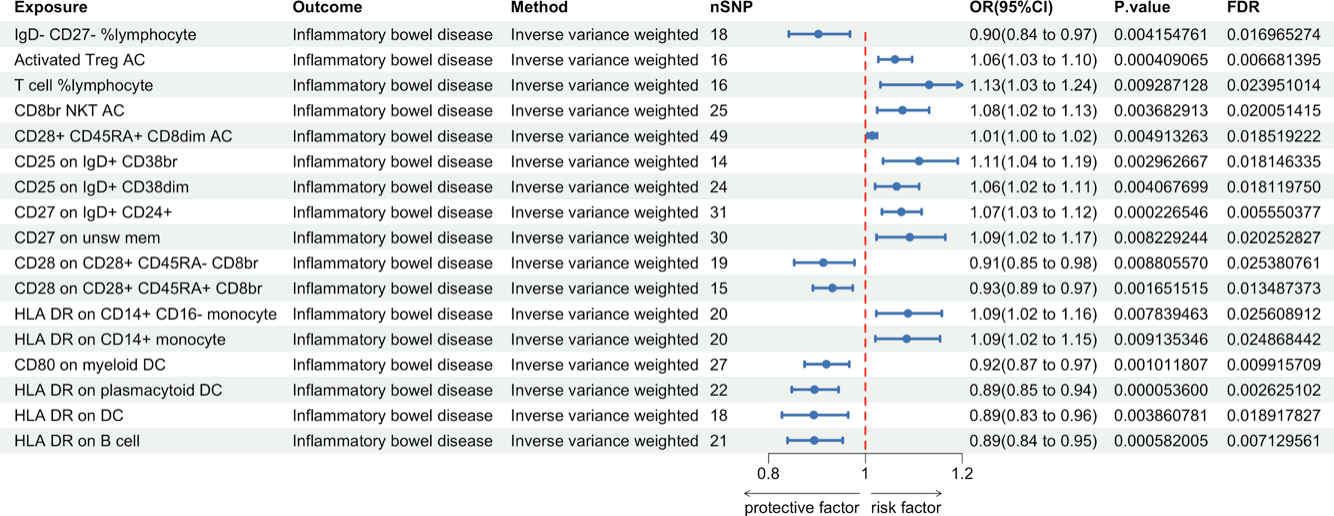
Causal effect of immune cells on IBD. CD = Crohn's disease, CI = confidence interval, FDR = false discovery rate, OR = odds ratio, SNP = single nucleotide polymorphism.

**Figure 3.**
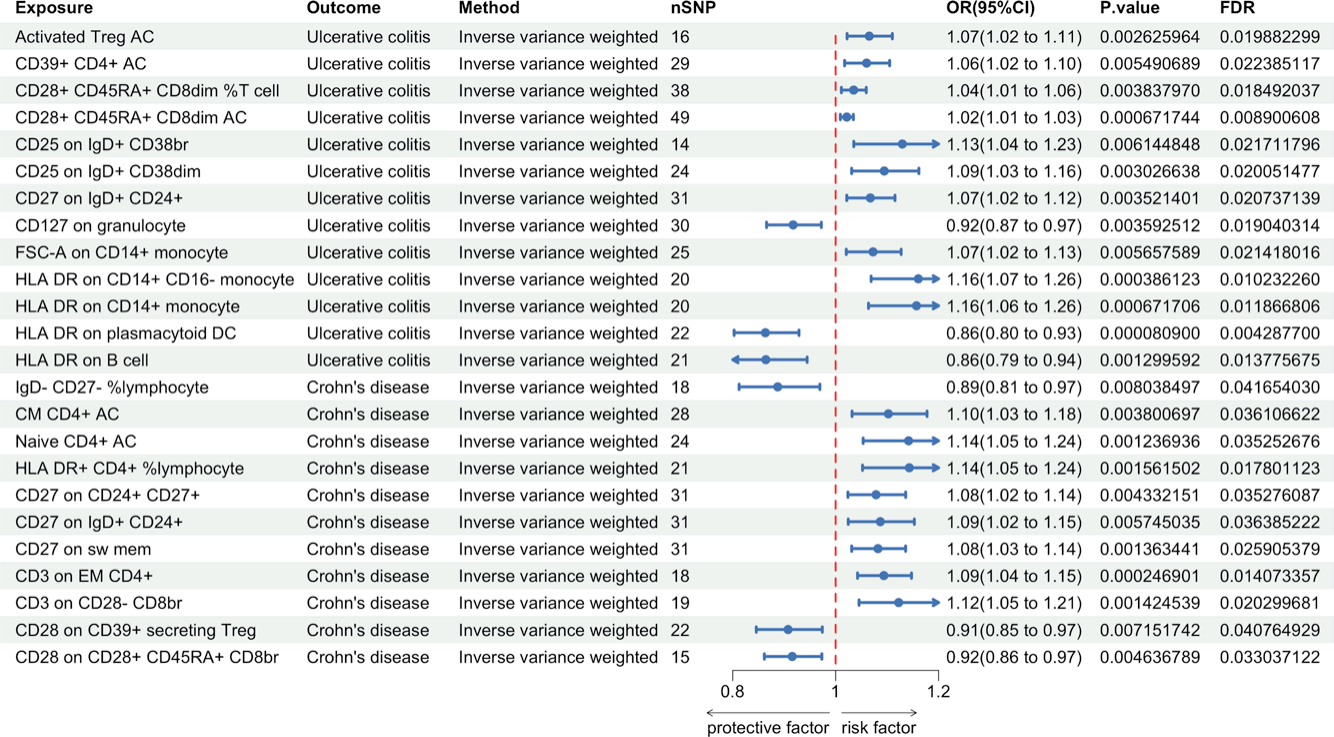
Causal effect of immune cells on UC and CD. CD = Crohn's disease, CI = confidence interval, FDR = false discovery rate, OR = odds ratio, SNP = single nucleotide polymorphism, UC = ulcerative colitis.

MR analysis encompassed 731 immune cells and their associations with IBD (including subtypes, UC and CD). IVW results demonstrated significant causal associations between 17 immune cells and IBD, 13 immune cells and UC, and 11 immune cells and CD (*P* < .01, FDR < 0.05). The MR-Egger intercept test and Cochran’s *Q* test indicated no pleiotropy or heterogeneity. LOO testing validated the stability of IVW results, as sequential removal of each SNP did not significantly affect the causal estimates, validating the robustness of the results (Figs. 2, 3, Tables S1, S9, and S17, Supplemental digital Content, https://links.lww.com/MD/Q766).

### 3.2. MR analysis of plasma metabolites and IBD and its subtypes

**Figure 4.**
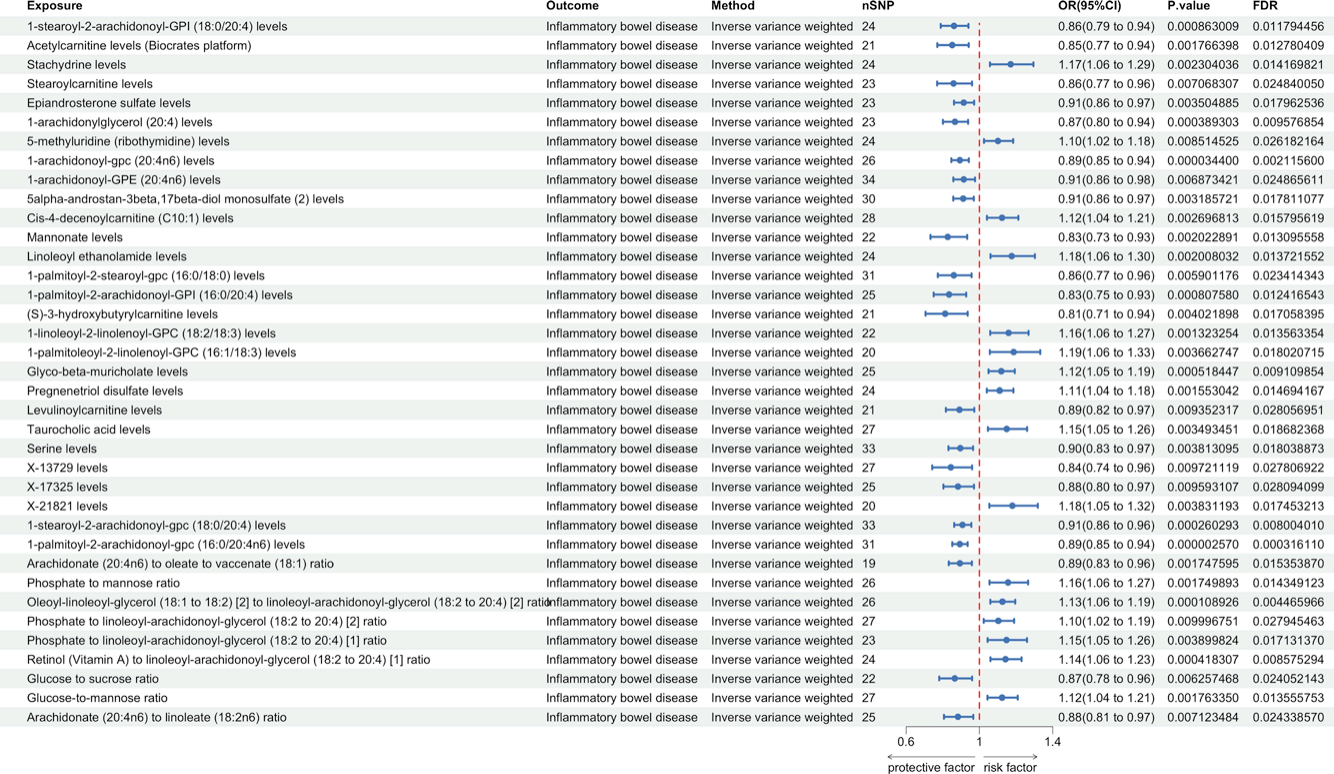
Causal effect of plasma metabolites on IBD. CD = Crohn's disease, CI = confidence interval, FDR = false discovery rate, OR = odds ratio, SNP = single nucleotide polymorphism.

**Figure 5.**
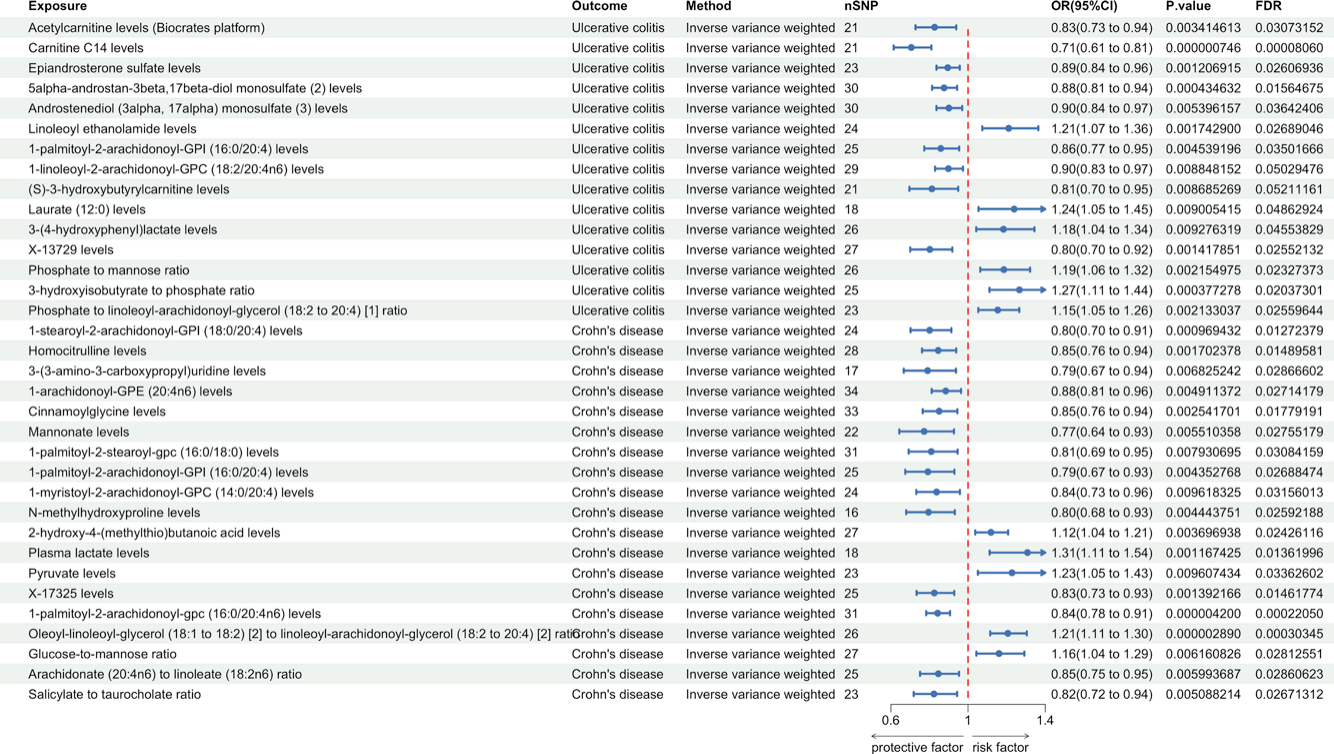
Causal effect of plasma metabolites on UC and CD. CD = Crohn's disease, CI = confidence interval, FDR = false discovery rate, OR = odds ratio, SNP = single nucleotide polymorphism, UC = ulcerative colitis.

A total of 1400 plasma metabolites were analyzed in relation to IBD and its subtypes UC and CD in MR analysis. IVW analysis identified 37 plasma metabolites significantly associated with IBD, 13 with UC, and 19 with CD (*P* < .01, FDR < 0.05). MR-Egger regression assessed the horizontal pleiotropy, with intercepts >0.05, indicating no horizontal pleiotropy. Cochran’s *Q* test assessed SNP heterogeneity, with *Q*_*P* values > 0.05 for both IVW and MR-Egger tests, indicating no heterogeneity. The LOO test further validated the robustness of the results (Figs. 4, 5, Tables S2, S10, and S18, Supplemental digital Content, https://links.lww.com/MD/Q766).

### 3.3. Mediation analysis results

**Figure 6.**
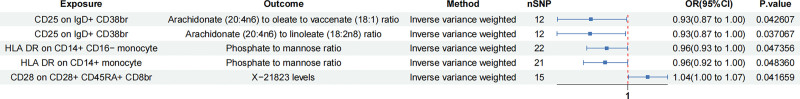
Causal effect of immune cells on plasma metabolites (IBD). CD = Crohn's disease, CI = confidence interval, IBD = inflammatory bowel disease, OR = odds ratio, SNP = single nucleotide polymorphism.

**Figure 7.**
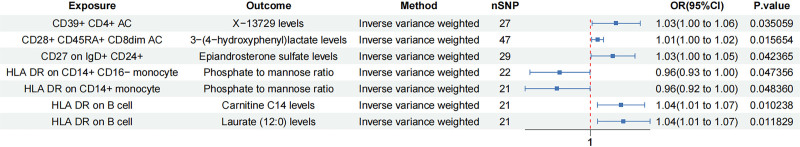
Causal effect of immune cells on plasma metabolites (UC). CD = Crohn's disease, CI = confidence interval, OR = odds ratio, SNP = single nucleotide polymorphism, UC = ulcerative colitis.

**Figure 8.**
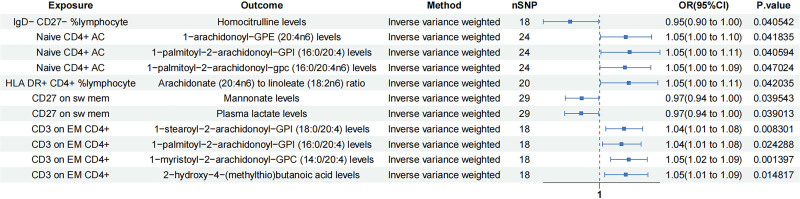
Causal effect of immune cells on plasma metabolites (CD). CD = Crohn's disease, CI = confidence interval, OR = odds ratio, SNP = single nucleotide polymorphism.

**Figure 9.**
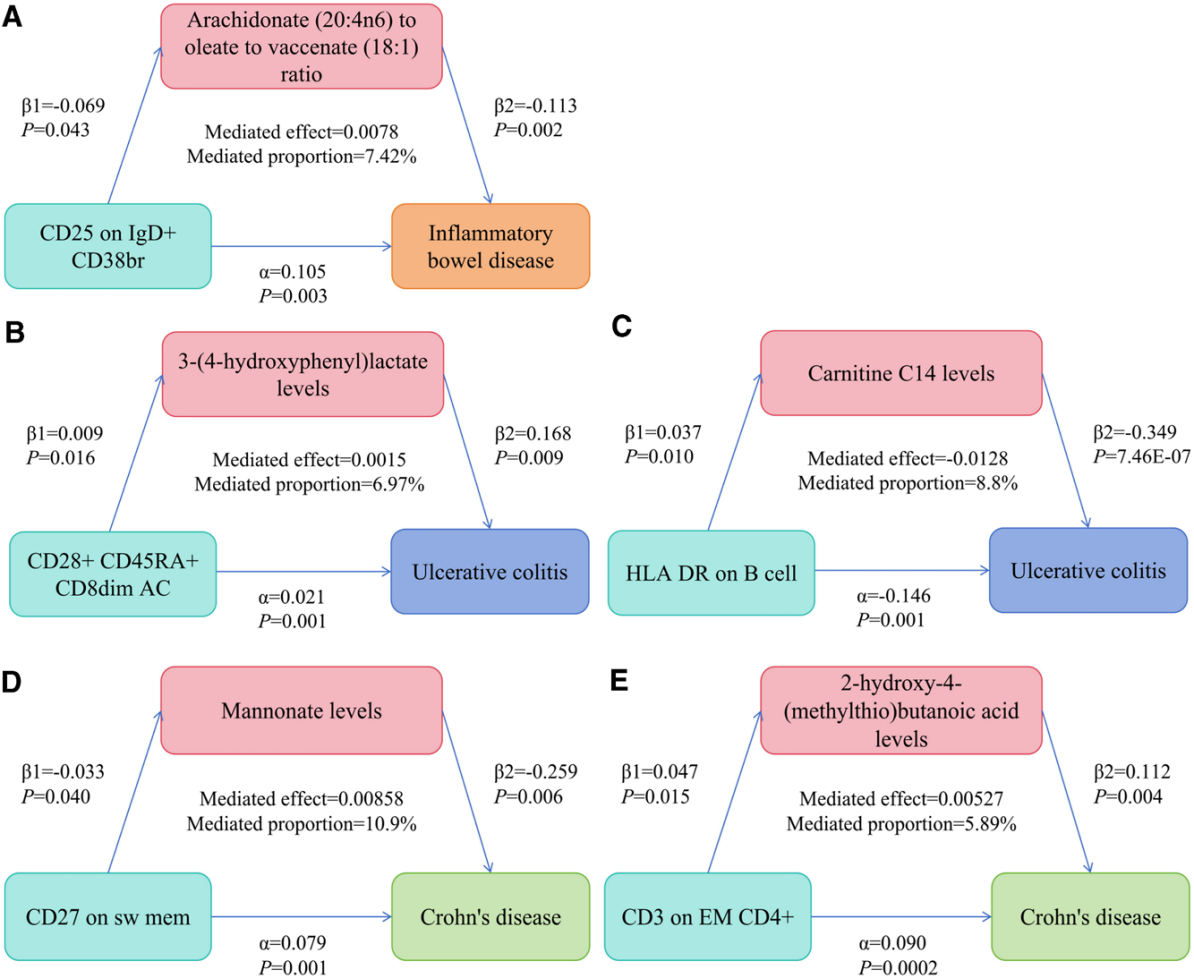
Mediation analysis schematic. (A) The association between CD25 on IgD+ CD38br and IBD was mediated by the Arachidonate (20:4n6) to oleate to vaccenate (18:1) ratio. (B) The association between CD28+ CD45RA+ CD8dim AC and UC was mediated by the 3-(4-hydroxyphenyl) lactate levels. (C) The association between HLA DR on B cell and UC was mediated by the Carnitine C14 levels. (D) The association between CD27 on sw mem and CD was mediated by the Mannonate levels. (E) The association between CD3 on EM CD4+ and CD was mediated by the 2-hydroxy-4-(methylthio) butanoic acid levels. CD = Crohn's disease.

TSMR assessed the role of plasma metabolites in mediating the effects of immune cells on IBD. Initially, MR analysis identified associations among immune cells and plasma metabolites: 5 immune cells correlated with 4 plasma metabolites in IBD, 6 immune cells with 6 plasma metabolites in UC, and 5 immune cells with 10 plasma metabolites in CD (Figs. 6–8, Tables S7, S15, and S23, Supplemental digital Content, https://links.lww.com/MD/Q766). Subsequent mediation analysis revealed that the association between CD25 on IgD+ CD38br and IBD was mediated by the arachidonate (20:4n6) to oleate to vaccenate (18:1) ratio, with a mediation proportion of 7.42%. CD28+ CD45RA+ CD8dim AC and HLA DR on B cells mediated the association between 3-(4-hydroxyphenyl) lactate, carnitine C14 levels and UC, with mediation proportions of 6.97% and 8.8%, respectively. CD27 on sw mem and CD3 on EM CD4+ mediated the association between mannonate levels, 2-hydroxy-4-(methylthio) butanoic acid levels, and CD, with mediation proportions of 10.9% and 5.89% (Table 2, Fig. 9, Tables S8, S16, and S24, Supplemental digital Content, https://links.lww.com/MD/Q766).

**Table 2:** MR analysis of causal associations of immune cells and plasma metabolites with IBD and its subtypes.

Exposure	Mediator	Outcome	Mediated effect (95% CI)	Mediated proportion (95% CI)	*P*-value
CD25 on IgD+ CD38br	Arachidonate (20:4n6) to oleate to vaccenate (18:1) ratio	Inflammatory bowel disease	0.0078 (−0.00106, 0.0167)	7.42% (−1.01%, 15.8%)	.084
CD28+ CD45RA+ CD8dim AC	3-(4-Hydroxyphenyl) lactate levels	Ulcerative colitis	0.0015 (0.000281, 0.00271)	6.97% (1.31%, 12.6%)	.016
HLA DR on B cell	Carnitine C14 levels	Ulcerative colitis	−0.0128 (−0.0227, −0.00298)	8.8% (15.6%, 2.04%)	.011
CD27 on sw mem	Mannonate levels	Crohn’s disease	0.00858 (0.000344, 0.0168)	10.9% (0.434%, 21.3%)	.041
CD3 on EM CD4+	2-Hydroxy-4-(methylthio) butanoic acid levels	Crohn’s disease	0.00527 (0.000677, 0.00987)	5.89% (0.755%, 11%)	.025

CD = Crohn's disease, CI = confidence interval, IBD = inflammatory bowel disease, MR = Mendelian randomization.

## 4. Discussion

This study examined the effects of 731 immune cells on IBD, UC, and CD through 1400 plasma metabolites, employing numerous publicly available genetic data. It is the inaugural MR analysis exploring the causal associations between multiple immune cells and IBD, UC, and CD, mediated by plasma metabolites. The analysis suggests a potential link involving 17 immune cells and 37 plasma metabolites with IBD; 5 immune cells affected IBD via 4 plasma metabolites. In UC, 13 immune cells and 15 plasma metabolites were implicated, with 6 immune cells exerting effects through 6 plasma metabolites. For CD, 11 immune cells and 19 plasma metabolites showed potential link, where 5 immune cells influenced CD through 10 plasma metabolites. Specifically, the association between CD25 on IgD+ CD38br and IBD was mediated by the arachidonate (20:4n6) to oleate to vaccenate (18:1) ratio. The positive association between CD28+ CD45RA+ CD8dim AC and UC was mediated by 3-(4-hydroxyphenyl) lactate levels, while the inverse association between HLA DR on B cells and UC was mediated by carnitine C14 levels. The positive association of CD27 on sw mem and CD3 on EM CD4+ (ORIVW = 1.094) with CD was mediated by mannonate level and 2-hydroxy-4-(methylthio) butanoic acid level, respectively.

CD25 on IgD+ CD38br and CD27 on sw mem are part of the B cell panel. B cells, pluripotent stem cells originating from the bone marrow, play a pivotal role in acquired immunity.^[25]^ The differentiation process of mammalian B cells can be divided into 5 stages: pro-B cells, immature B cells, mature B cells, activated B cells, and plasma cells. Within the mature B cell stage, cells are further classified into Bm1–Bm5 subsets based on expression patterns of CD38 and IgD. CD25+ IgD+ CD38 cells correspond to the Bm2 stage. CD25, the α chain of the IL-2 receptor (IL-2R), is crucial for cell growth, survival, differentiation, and immunity.^[26]^ Contemporary research indicates that IL-2 orchestrates both immunostimulatory and immunosuppressive functions by activating distinct target cells. As a pivotal regulator of immune homeostasis, dysregulated IL-2 expression is implicated in multiple immune-related disorders.^[27,28]^ Elevated frequencies of hiCD25+ cells are commonly observed in T cells from CD patients and macrophages in UC, both subtypes of IBD.^[29]^ CD27 (TNFRSF7), is a transmembrane glycoprotein constitutively expressed on CD4+/CD8+ T cells, NK cells, and thymocytes, with induced expression upon B cell activation. Consequently, it serves as a canonical marker for human memory B cells.^[30,31]^ Through bioinformatic analysis and immunohistochemical evaluation of CD tissues, Yu et al observed elevated CD27+ B cell densities compared to normal intestinal mucosa, revealing a positive correlation between CD27 expression and CD activity indices.^[32]^ Notably, CD27 belongs to the tumor necrosis factor receptor superfamily (TNFRSF) – a group of proteins orchestrating immune and inflammatory responses. In CD pathogenesis, TNF-α is frequently upregulated. Infliximab, a TNF inhibitor, serves as a therapeutic agent for IBD and is fundamental in treating moderate to severe UC and CD.^[33]^

CD28+ CD45RA+ CD8dim AC is included in the Treg panel; and CD3 on EM CD4+ is within the T cell panel. CD3 on EM CD4+ refers to cells that express the CD3 molecule on the surface of CD4+ T cells. T cells, derived from lymphoid stem cells in the thymus, constitute the most abundant and functionally diverse lymphocyte population. There are 2 main types of T cells in the human body, one of which is CD4+ T cells. These cells carry the CD4 protein marker on their surface and are also known as helper T (Th) Cells. Their primary functions include activating B cells, regulating tissue homeostasis, mediating immune modulation, and playing central roles in combating infections, antitumor responses, and immune regulation. CD4+ T cells can further differentiate into functional subtypes such as Th1, Th2, Th17, Treg (regulatory T cells), and Tfh (follicular helper T cells).^[34]^ Treg cells typically exert negative regulation on immune functions, while helper Th17 cells mediate immune responses, maintaining a dynamic equilibrium. A reduction in Treg cells or their dysfunction hampers the suppression of Th17 cell-induced inflammation, leading to elevated inflammatory factors and damage to the intestinal mucosal barrier, potentially triggering UC.^[35]^ CD3, an activation signal molecule, transduces TCR signals to T cells. Røyset ES et al demonstrated, using deep learning-based whole slide images, that the number of CD3 cells in colon mucosal tissue was higher in inactive CD patients compared to normal and inactive UC groups.^[36]^ CD28 is a transmembrane glycoprotein expressed on the T-cell surface, functioning as a co-stimulatory receptor for the T-cell receptor. Upon binding to its ligands (CD80/CD86), CD28 delivers co-stimulatory signals that amplify T-cell-mediated immune responses. As a key immunoregulator in autoimmune diseases, CD28 overexpression has been reported in systemic lupus erythematosus, systemic sclerosis, and related conditions, correlating with disease pathogenesis.^[37–39]^ HLA DR on B cells belong to the TBNK panel. HLA-DR is a major histocompatibility complex class II antigen-presenting molecule, and predominantly expressed on antigen-presenting cells such as macrophages, dendritic and B cells, and is crucial for lymphocyte activation and immune regulation.^[40,41]^ Reduced HLA-DR expression impairs antigen-presenting capacity and compromises adaptive immune responses. In IBD, HLA alleles constitute major genetic risk factors. Notably, research reveals that elevated HLA-DR expression on CD14+ monocytes is associated with reduced IBD susceptibility.^[42]^

This study identified the arachidonate (20:4n6) to oleate to vaccenate (18:1) ratio, 3-(4-hydroxyphenyl) lactate levels, carnitine C14 levels, mannonate levels, and 2-hydroxy-4-(methylthio) butanoic acid levels as mediation metabolites for IBD, UC, and CD. Arachidonate (AA, 20:4n6), oleate, and vaccenate (18:1) are fatty acids, with AA being a key polyunsaturated fatty acid and an essential component of cell membranes. polyunsaturated fatty acid modulates intestinal inflammation, influencing IBD.^[43]^ Elevated AA levels have been observed in the UC patients’ colonic mucosa, with a positive correlation between dietary AA intake and UC risk.^[44]^ Oleate and vaccenate (18:1), as monounsaturated fatty acids, have shown protective effects on intestinal epithelial cells in CD patients.^[45]^ The 3-(4-hydroxyphenyl) lactate, a decomposition product of lactic acid bacteria in phenylalanine, is closely linked to IBD pathogenesis. Phenylalanine levels significantly differ between UC and CD patients and healthy individuals, indicating its potential as a biomarker for diagnosing IBD.^[46,47]^ UC patients frequently exhibit impaired fatty acid oxidation, with carnitine essential for transporting fatty acids into mitochondria for oxygenolysis.^[48]^ Previous studies have demonstrated the efficacy of propionyl-l-carnitine hydrochloride in treating mild to moderate UC.^[49]^ mannonate, linked to glucose metabolism, can disrupt immune homeostasis and exacerbate inflammation.^[50]^ The 2-hydroxy-4-(methylthio) butanoic acid (a precursor of the essential amino acid methionine) is converted to methionine and subsequently hydrolyzed to homocysteine (Hcy) via methionine adenosyltransferase and methyltransferases.^[51,52]^ In IBD, the inhibition of Hcy remethylation and transsulfuration pathways leads to elevated plasma Hcy levels.^[53]^ Hyperhomocysteinemia can exacerbate intestinal mucosal injury and inflammation through oxidative stress, inflammation, and endothelial dysfunction, thus contributing to IBD progression.^[54,55]^

A 2-sample MR analysis based on data from a published large-scale GWAS cohort, encompassing 1,50,000 individuals with high statistical power, was utilized in this study. The results, derived from genetic IV and causal inference using various MR methods, ensure a reliable and comprehensive assessment of the strong association between immune cells, plasma metabolites, and IBD, including its subtypes. However, it has several limitations. Although multiple sensitivity analyses are performed, it does not fully address horizontal pleiotropy among immune cells, plasma metabolites, and IBD. Furthermore, immune cell datasets may lack resolution, and certain metabolite alterations may represent disease biomarkers rather than causal drivers of pathogenesis. Additionally, the absence of individual data precludes stratified population analysis. The reliance on a European database also limits the generalizability of the results to other populations. Further validation through comprehensive clinical trials is necessary to obtain more precise clinical conclusions. Expanding the GWAS database and employing additional analysis methods or experimental verification are essential to elucidate the association of immune cells with IBD and related mediation mechanisms via plasma metabolites.

## 5. Conclusion

This MR analysis demonstrates a causal association of immune cells and plasma metabolites with IBD and its subtypes. Mediation analysis indicates that plasma metabolites mediate the association between immune cells and IBD, including its subtypes. These insights provide new perspectives on the mechanisms driving IBD development and potential diagnostic and therapeutic targets.

## Acknowledgments

We thank all GWAS participants, researchers, and authors for their contributions.

## Author contributions

**Conceptualization:** Na Wu.

**Data curation:** Zhong Li, Haiyan Wen, Xueyun Huang.

**Funding acquisition:** Na Wu.

**Software:** Zhong Li, Haiyan Wen, Xueyun Huang.

**Writing – original draft:** Zhong Li, Haiyan Wen.

**Writing – review & editing:** Na Wu.

## Supplementary Material


